# Dr. Jackson's Remarks on Cobweb

**Published:** 1809-11

**Authors:** Robert Jackson

**Affiliations:** Castle Eden, Durham


### *Dr.* JACKSON'*s Remarks on Cobweb.*

To the EDITORS of the MEDICAL and PHYSICAL JOURNAL.

GENTLEMEN,

AS my account of the virtues of Cobweb, inserted in the Medical Journal of last May, is discredited by a person who signs himself an *Essex Practitioner,* you will be so obliging as to give the following Observations a place in your next publication. The Essex Practitioner, as not giving his name to his communication, deserves no notice on his own account; but, as cobweb is a remedy of value, I am desirous that it should be known generally, and that its character should be established publicly on sure grounds. Its power in stopping the course of intermittents does not rest on my authority alone ; it is known to many as possessing that quality ; and among others, a Lady who lives near the mouth of the Tees, on the Durham side of the river, in a situation where agues prevail in some seasons, has, in the course of her life, cured hundreds by means of this substance, which the Essex Practitioner terms inert. She does not prepare the subject previously to the exhibition of the remedy ; and, as might be expected, she does not always succeed ; but she often succeeds where the regular practitioner fails. She has not noticed the circumstances which mar its successful action, nor shall I at present attempt to define them, but simply state the facts. Cobweb was giving by her lately to two persons who were in the same house ; one was perfectly cured ; the other was not in the least benefited. Five were cured in one week ; besides others in the course of the season, some of whom were extremely reduced and much wasted by the disease, which had obstinately resisted bark and the ague drop. She mixes the cobweb with treacle, and gives it in large quantity, perhaps to the size of a nutmeg. The person does not know what is given to him, consequently, imagination has nothing to do in the case. Relapse of intermittents cured in this manner is rare.—The Lady is a person of respectability, and though she would not, probably, chuse that her name should appear in the Journal, she would not, I believe, refuse her testimony to what is now stated, if any one should be disposed to doubt the truth of it.

That cobweb is capable of stopping the course of intermittents, is as well proved as any fact in physic; but it is also true, that it acts powerfully in all the other conditions noticed in my paper of May; and I may add, in many others that I omitted to mention. I shall trouble you with only one testimony. In the month of last June, an army physician, who exercises his profession in civil life, and to whom I was a little known, accosted me, and took the opportunity of mentioning that he had employed cobweb in a troublesome case, in consequence of what I had said of it in the Medical Journal. The Case, he observed, was one of pain, distress, and watchfulness, of unusual obstinacy. The cobweb was ordered, faithfully prepared, and administered. The effect appeared to have surprized the patient; for he called upon his physician next morning to say, (if I recollect the words right, they were spoken in the street) That he had not passed a night with so much comfort for six years. The remedy was repeated; and it always gave ease and tranquillity, though the patient did not always sleep. The patient, who was a gentleman, left the place, and the physician thought it his duty to let him know the means that had afforded him so much relief. The physician is a respectable man, and though he might not chuse that his name should appear in the Journal, as employing so vulgar a remedy as cobweb, I do not suppose that he would refuse to state to the Editor, or other creditable person, the circumstances of the case to which I allude.

I shall add no more on the subject at present; the Essex Practitioner will, I doubt not, be sorry that he has committed himself prematurely.

I am, &c.

ROBERT JACKSON, M. D.

*Castle Eden, Durham, Sept.* 29, 1809.

---

### *To the Editors of the Medical and Physical Journal.*

"Who shall decide, when Doctors disagree,
"And soundest casuists doubt, like you and me?" Pope.

Gentlemen,

